# Serum high-density lipoprotein correlates with serum apolipoprotein M and A5 in obstructive sleep apnea hypopnea syndrome

**DOI:** 10.1007/s11325-016-1357-5

**Published:** 2016-05-21

**Authors:** Shengyu Tan, Xianling Liu, Yan Xu, Lu Luo, Shenghua Zhou, Yawen Gao

**Affiliations:** 1 Department of Gerontology, The Second Xiangya Hospital, Central South University, Changsha, 410011 China; 2 Department of Oncology, The Second Xiangya Hospital, Central South University, Changsha, 410011 China; 3 Department of Cardiovascularology, The Second Xiangya Hospital, Central South University, Changsha, 410011 China

**Keywords:** Obstructive sleep apnea hypopnea syndrome, High-density lipoprotein, Apolipoprotein M, Apolipoprotein A5, Nasal continuous positive airway pressure treatment

## Abstract

**Purpose:**

The purpose of this study was to investigate the correlation between serum levels of serum apolipoprotein M (ApoM), A5 (ApoA5), and high-density lipoprotein (HDL) in patients with obstructive sleep apnea hypopnea syndrome (OSAHS) and study the effects of nasal continuous positive airway pressure treatment on these serum biomarkers.

**Methods:**

Thirty OSAHS patients and 15 non-OSAHS probands as control were selected for the study. Serum HDL, ApoM, and ApoA5 levels in two groups were detected; differences and association among them were analyzed. Patients with moderate and severe OSAHS underwent 3-month auto-continuous positive airway pressure treatment, and a comparative study was conducted to investigate the changes in blood lipids, serum ApoM, and ApoA5.

**Results:**

In comparison to the control group, the HDL, ApoM, and ApoA5 serum levels were lower (*P* < 0.05). HDL was positively correlated to ApoM and ApoA5 (*P* < 0.001), and ApoM was positively correlated to ApoA5 (*r* = 0.536, *P* < 0.001). HDL, ApoM, and ApoA5 were significantly increased in the patients of moderate and severe OSAHS after auto-continuous positive airway pressure treatment for 3 months (*P* < 0.05).

**Conclusions:**

The HDL level was significantly lower in OSAHS patients. The decrease in serum ApoM and ApoA5 in OSAHS patients was correlated to the severity of OSAHS and HDL levels. Auto-continuous positive airway pressure treatment increased serum levels of ApoM, ApoA5, and HDL in OSAHS patients.

## Introduction

Obstructive sleep apnea hypopnea syndrome (OSAHS) is a disorder with a high incidence and potential risks, manifested as snoring accompanied with respiratory pauses, hypopnea, shallow breathing, recurrent hypoxemia, and hypercapnia acidosis during the night [1–3]. This sleep structure disorder might lead to daytime sleepiness and multiple organ damage, as well as seriously affecting quality of life [4].

The adverse effects of OSAHS include its high incidence and the involvement of multiple organs and systems [5–7], as well as its close association with cardiovascular diseases such as hypertension, coronary heart disease, and atherosclerosis [8, 9]. OSAHS is an important causative factor for coronary heart disease, independent from other factors such as age, obesity, smoking, diet, and genetic background. Lavi [10] and Svatikova [11] discovered that the increased incidence of cardiovascular disease in OSAHS patients might be related to their lipid metabolism disorder. Therefore, a thorough investigation of a possible correlation between OSAHS and the lipid metabolism disorder is needed. It has been shown that the blood lipid profile of OSAHS patients is abnormal, manifesting as a significant increase in triglyceride (TG), total cholesterol (CHOL), very low-density lipoprotein (VLDL), low-density lipoprotein (LDL), apolipoprotein B (ApoB), and lipoprotein a (Lpa) in serum, while high-density lipoprotein (HDL) and apolipoprotein A (ApoA) levels decrease. This abnormal blood lipid profile might be a causative factor of cardiovascular disease [12–14].

ApoA5 belongs to the apolipoprotein superfamily discovered in 2001 by Pennacchio et al. [15, 16]. The accumulated animal studies and epidemiological studies indicate that ApoA5 is closely connected with lipid metabolism. ApoA5 causes a decrease in the serum levels of TG and is considered as a negative regulator of TG [15]. ApoA5 was reported to be associated with HDL, VLDL, and chylomicron in human serum [17]. Furthermore, it was also suggested that ApoA5 produced in liver tissue could promote ApoA1 elevation in the serum, resulting in increased serum levels of HDL-C. ApoA5 also enhances cholesterol release from macrophages [18]. The lecithin cholesterol acyltransferase (LCAT), which plays a vital role in the maturation of HDL, was reported to be positively associated with ApoA5 [19]. All these findings suggest that ApoA5 might promote HDL maturation, thus regulating the anti-atherosclerosis function of HDL by manipulating the size and subgroups of HDL in the serum.

Apolipoprotein M (ApoM) is a newly discovered apolipoprotein which has a special structure and is considered to play an important role in the metabolism of HDL and the anti-atherosclerosis process [20]. HDL biosynthesis consists of a series of complicated linked reactions, with various apolipoproteins involved. The mouse model study by Wolfrum et al. [21] showed that the expression of ApoM in liver was reduced by 90 % through RNAi against ApoM mRNA, which resulted in a significant decrease of ApoM in serum and a 25 % decrease of serum HDL-C.

OSAHS and hyperlipidemia are common but typically neglected by patients. The correlation between these two conditions is still in early stages of evaluation. ApoM and ApoA5 are apolipoproteins which have been gradually investigated in the recent years; however, clinical research documenting the relationship between the levels of ApoM, A5, and HDL-C in the serum of OSAHS patients has not been published yet. Therefore, this study aimed at measuring the serum levels of ApoM, A5, and HDL in OSAHS patients and investigating the influence of nasal continuous positive airway pressure treatment on ApoM and ApoA5.

## Materials and methods

### Patients

This was a retrospective study of 45 patients under examination by polysomnography (PSG) in the sleep laboratory of the Second Xiangya Hospital of Central South University in China, between January 2013 and October 2013. Among the 45 subjects, 30 were patients with mild, moderate, and severe OSAHS (Apnea Hypopnea Index (AHI) ≥ 5), as well as additional 15 probands as controls (AHI < 5).

AHI measures the number of apnea and hypopnea events per hour of sleep. The apneas lasting at least 10 s are associated with a decrease in blood oxygenation. The OSAHS was diagnosed based on the following manifestations: recurrent apnea and hypopnea occurring more than 30 times during the night lasting 7 h, AHI > 5, obstructive apnea accompanied with snoring, sleep apnea, and daytime sleepiness. The 5 ≤ AHI <15 range of values was defined as mild OSAHS, the 15 ≤ AHI <30 range was defined as moderate OSAHS, and AHI ≥ 30 was defined as severe OSAHS [22].

Exclusion criteria for all subjects included a history of smoking or alcoholism (no drinking history or drinking <140 g/week for men and <70 g/week for women was allowed); myocardial infarction, unstable angina pectoris, valvular heart disease, cardiomyopathy, severe arrhythmia, stroke, tumor, severe renal insufficiency, severe lung diseases, infections, trauma, or surgeries 2 weeks prior to the study.

For patients receiving medications, oral antihypertensive agents were continued, including amlodipine and perindopril in patients with high blood pressure; there were no oral medications being supplied to the patients with diabetes.

Written informed consent was obtained from all subjects before the study and the study was performed in accordance with the ethical standards laid down in the 1964 Declaration of Helsinki and its later amendments. The research protocol was approved by the Ethics Committee of Department of Geriatric at the Second Xiangya Hospital, Central South University in China.

### General information collection

General information, including disease history, family history, smoking or drinking habits, and medication history during the recent 6 months was collected through questionnaires. Physical measurements such as height, weight, waist circumference (WC), hip circumference (HC), neck circumference (NC), systolic blood pressure (SBP), diastolic blood pressure (DBP), body mass index (BMI), and waist hip ratio (WHR) were also archived for analysis.

### Polysomnography

The sleep patterns of patients were monitored by digital polysomnography (REMBRANDT, Medcare, Buffalo, NY, USA) for more than 7 h during the night; meanwhile, electroencephalogram (EEG), electromyogram (EOG), electromyography (EMG), electrocardiogram (ECG), snoring, oronasal airflow and thoracoabdominal motion, and fingertip oxygen saturation (nocturnal mean and minimum SaO_2_) were also recorded.

The results from polysomnography recordings, including the following parameters: AHI, total sleep apnea time, sleep low ventilation time, the longest sleep respiratory pause time, lowest oxygen saturation (LSaO_2_), mean oxygen saturation (MSaO_2_), the percentage of blood oxygen saturation below 90 % during sleep (T-SaO_2_ < 90 %), night awakening times and duration, were all computer-analyzed and curated manually before releasing the data.

### Continuous positive airway pressure

The patients with moderate to severe OSAHS received a therapeutic trial of auto-continuous positive airway pressure (RESMED, Sydney, Australia) during the next 3 months.

### Collection and storage of serum samples

Blood samples were obtained from each participant after the PSG test. They were obtained once again from patients after the 3-month auto-continuous positive airway pressure treatment. Serum from 3 mL of venous blood, drawn from the ulnar vein, the supernatant was stored in the −70 °C freezer after 10 min of centrifugation at a speed of 3000 rpm.

### Serum samples measurement

Serum levels of CHOL, TG, HDL-C, and LDL-C were measured using the automatic biochemical analyzer, type 7600 (Hitachi, Tokyo, Japan).

ELISA kits (R&D Systems, Minneapolis, MN, USA) were used in the current study for determination of serum ApoM and ApoA5 levels. Briefly, the serum sample and biotinylated antibody were added to ELISA plates sequentially and washed by PBS or TBS. Subsequently, the antibody-antigen complex was recognized using streptavidin labeled peroxidase and visualized by TMB after thoroughly washing with BS or TBS. TMB turned blue under peroxidase treatment and later changed to yellow under acidic conditions. The color depth reflects the concentration of the tested substrate; the darker the color, the higher the concentration. The automatic ELISA microplate reader (Rayto Life and Analytical Sciences Co., Ltd. Shenzhen, China) was utilized for quantitation.

### Statistical analysis

SPSS13.0 software was utilized in this study. Quantitative data fitting a normal distribution pattern were presented as mean ± SD. The data which was not normally distributed was presented as the median and range. The data not fitting a normal distribution were subjected to natural logarithm operation or geometric mean calculation before statistical analysis. The *t* test and variance analysis were used to compare data means, while the *χ* ^2^ test was utilized for ratio comparison. The HDL-C, ApoM, and ApoA5 levels in controls and patients with different severity of OSAHS were analyzed by Kruskal-Wallis test. The multivariate stepwise regression analysis was adapted for multiple factors’ analysis, with *P* < 0.05 considered as statistically significant.

## Results

### Clinical information

There were no significant differences between the two studied groups in terms of age, gender, BMI, and disease distribution. The neck circumference, the waist hip ratio, the AHI, and the serum level of TG were higher in the OSAHS group compared with controls. Serum levels of HDL, ApoM, and ApoA5 were lower in the OSAHS group, and the mean nocturnal SaO_2_ and minimum SaO_2_ were significantly lower in the OSAHS group in comparison to the controls. There was no significant difference between the two groups with respect to serum levels of CHOL and LDL-C (Table 1).

**Table 1:** General information of OSAHS and control groups

	OSAHS group (*n* = 30)	Control group (*n* = 15)	*P*
Gender (man:woman)	23:07	12:03	NS
Age (year)	38 ± 15	39 ± 14	0.746
BMI (kgW)	27 ± 3	26 ± 2	0.153
Neck circumstance (m)	0.58 ± 0.06	0.38 ± 0.03	0.004*
Waist hip ratio	0.96 ± 0.04	0.86 ± 0.06	0.001*
Complicated with hypertension (case)	6	3	NS
Complicated with diabetes (case)	4	1	NS
AHI	30 ± 18	3 ± 1	<0.0001*
Mean SaO_2_ (%)	91 ± 2.09	96 ± 2.53	<0.001*
Minimum SaO_2_ (%)	68 ± 18.17	88 ± 5.49	<0.0001*
CHOL (mmol/L)	4.36 ± 1.12	4.38 ± 1.38	0.325
TG (mmol/L)	2.17 ± 1.03	1.48 ± 0.55	0.025*
HDL-C (mmol/L)	0.88 ± 0.29	1.56 ± 0.27	0.015*
LDL-C (mmol/L)	2.50 ± 0.75	2.49 ± 0.83	0.684
ApoM (pg/mL)	921.05 ± 382.33	1708.81 ± 1584.38	0.027*
ApoA5 (pg/mL)	3221.31 ± 62.51	5776.11 ± 94.3	0.028*

*P < 0.05 indicates statistically significant difference

### The relationship between OSAHS severity and ApoM and ApoA5 serum levels

**Fig. 1:**
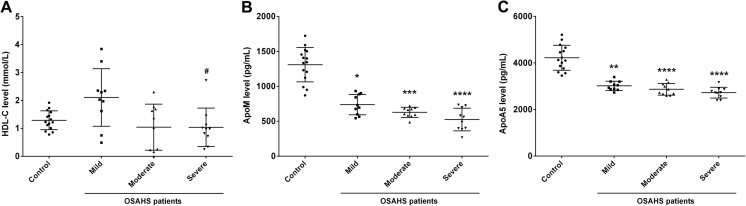
HDL-C, ApoM, and ApoA5 levels in healthy probands and patients with different severity of OSAHS. **a** HDL-C, **b** ApoM, and **c** ApoA5 levels in subjects. ^#^ *P* < 0.05 vs. mild OSAHS patients. *P < 0.05; **P < 0.01; ***P < 0.001; ****P < 0.0001 vs. healthy probands

The AHI was positively correlated to TG and negatively correlated to HDL, ApoM, and ApoA5 in all participants. The HDL-C level was significantly lower in severe OSAHS than in mild OSAHS (*P* < 0.05) (Fig. 1a) and the ApoM and ApoA5 levels were both significantly lower in each severity of OSASHS patients than in the healthy controls (*P* < 0.05) (Fig. 1b, c). The mean nocturnal SaO_2_ and minimum SaO_2_ were negatively correlated to serum levels of TG in all participants; whereas, the mean nocturnal SaO_2_ and minimum SaO_2_ were positively correlated to serum levels of HDL, ApoM, and ApoA5 (Table 2).

**Table 2:** Correlations between serum parameters

	AHI		Mean SaO_2_		Minimum SaO_2_		ApoM		Apo-A5	
	*r*	*P*	*r*	*P*	*r*	*P*	*r*	*P*	*r*	*P*
TG	0.45	0.002*	−0.464	0.005*	−0.565	0.001*	0.494	0.000*	0.562	0.000*
CHOL	0.063	0.749	0.064	0.951	0.365	0.32	0.202	0.277	0.213	0.523
HDL	−0.566	0.000*	0.471	0.000*	0.477	0.000*	0.56	0.000*	0.483	0.000*
LDL	0.063	0.754	0.154	0.75	0.369	0.33	0.257	0.171	0.292	0.3
ApoM	−0.397	0.006*	0.421	0.015*	0.533	0.003*	0.536	0.000*		
ApoA5	−0.357	0.015*	0.38	0.041*	0.395	0.012*				

*P < 0.05 indicates statistically significant difference

### Correlations and multivariate stepwise regression analysis for ApoM, ApoA5, and the other parameters

A positive correlation was observed between serum AHI and TG in all patients, while negative correlations were observed between AHI and HDL-C, ApoM, and ApoA5 in all patients (*P* < 0.05). The serum level of TG was negatively correlated to the mean nocturnal oxygen saturation (SaO_2_) and minimum SaO_2_, while HDL, ApoM, and ApoA5 were positively correlated to the mean nocturnal oxygen saturation (SaO_2_) and minimum SaO_2_ (*P* < 0.05). HDL was positively correlated to ApoM and ApoA5 (*P* < 0.001), and ApoM was positively correlated to ApoA5 (*r* = 0.536, *P* < 0.001). The serum TG level was significantly reduced in patients with moderate and severe OSAHS after a 3-month treatment with auto-continuous positive airway pressure (*P* < 0.05), while HDL, ApoM, and ApoA5 were increased significantly (*P* < 0.05).

The multivariate stepwise regression analysis was performed with ApoM as the dependent variable and AHI, mean SaO_2_, minimum SaO_2_, HDL-C, and ApoA5 as independent variable. It was also performed with ApoA5 as the dependent variable and the AHI, mean SaO_2_, minimum SaO_2_, HDL-C, and ApoM as independent variables. A good correlation was found between ApoM and HDL-C, and mean SaO_2_ (*β* = 0.020, 95%CI = 0.380–0.612, *P* = 0.0003), and also between ApoA5 and HDL-C, and mean SaO_2_ (*β* = 0.502, 95%CI = 0.260–0.562, *P* = 0.000).

### The effects of auto-continuous positive airway pressure treatment on patients with moderate or severe OSAHS

Twenty patients with moderate or severe OSAHS all completed the 3-month auto-continuous positive airway pressure treatment. The treatment lasted more than 7 h every night. The symptoms of daytime sleepiness, fatigue, and night awakenings due to shortness of breath, dizziness, and dry mouth upon awakening were all significantly alleviated. The AHI, mean nocturnal SaO_2_, and minimum SaO_2_ were all improved significantly (*P* < 0.05). The serum level of TG was reduced significantly after auto-continuous positive airway pressure treatment (*P* < 0.05), while there was no significant change in the BMI, neck circumference, and waist hip ratio. The serum levels of CHOL and LDL improved after treatment, but the difference remained insignificant (Table 3).

**Table 3:** Serum parameters before and after auto-continuous positive airway pressure treatment in moderate and severe OSAHS

	Before treatment (*n* = 20)	After treatment (*n* = 20)	*P*
BMI (kgW)	27.74 ± 2.14	27.44 ± 2.26	0.133
Neck circumference (m)	0.55 ± 0.36	0.58 ± 0.03	0.425
Waist hip ratio	0.94 ± 0.25	0.91 ± 0.36	0.198
AHI	37.68 ± 14.10	8.09 ± 5.04	0.001*
Mean SaO_2_ (%)	91.14 ± 2.34	95.96 ± 2.42	0.012*
Minimum SaO_2_ (%)	66.29 ± 18.49	87.29 ± 5.12	0.042*
CHOL (mmol/L)	3.84 ± 0.43	3.39 ± 1.03	0.485
TG (mmol/L)	2.62 ± 1.31	1.26 ± 0.53	0.017*
HDL-C (mmol/L)	0.89 ± 0.44	1.35 ± 0.27	0.018*
LDL-C (mmol/L)	1.78 ± 0.65	1.69 ± 0.80	0.339
ApoM (pg/mL)	881.6 ± 291.47	1934.15 ± 1120.22	0.016*
ApoA5 (pg/mL)	104.97 ± 59.89	229.94 ± 109.49	0.013*

## Discussion

### The effect of OSAHS on blood lipid metabolism

Epidemiological studies show that OSAHS incidence in the 40–60-year age group ranges between 5 and 10 % [23]. A survey found that 9 % of women and 24 % of men aged between 30 and 60 years have experienced sleep apnea, while 2 % of women and 4 % of men are diagnosed with sleep apnea syndrome [24]. OSAHS can affect multiple organs, leading to functional and organ damage, an impaired quality of life and a shortened life span. It has also been determined that OSAHS is an independent risk factor for various systemic diseases [15, 16].

Lipid metabolism disorder refers to elevated serum lipid levels, including increased levels of CHOL, TG, and HDL-C, and a decreased level of LDL-C. The main influencing factors include diet, lifestyle, age, gender, obesity, as well as genetic and psychological factors. In the current study, there was no significant difference between two groups in terms of age and gender; therefore, the potential influence of these two parameters on lipid metabolism was excluded. Our findings indicate that serum TG and HDL-C levels are significantly different in the OSAHS and control groups (*P* < 0.05). Significantly increased TG level and significantly decreased HDL-C level were found in the OSAHS group compared to controls, which was consistent with an earlier study [25].

The AHI is the objective parameter used to classify the severity of OSAHS. Chronic intermittent hypoxia is thought to be a primary trigger for hypertension in OSAHS and is likely to be implicated in activating inflammatory pathways that result in many comorbidities of OSAHS [26, 27]. The severity of nocturnal hypoxia is reflected by both mean nocturnal SaO_2_ and minimum SaO_2_ values. In the present study, the increased serum TG level was positively correlated with the AHI and negatively correlated with nocturnal mean SaO_2_ and minimum SaO_2_. On the other hand, the decreased serum HDL-C was negatively correlated with the AHI and positively correlated with the mean nocturnal SaO_2_ and minimum SaO_2_. This implies that more severe OSAHS has more significant lipid metabolism disorder. Accumulated studies indicate that OSAHS patients often present with hyperlipidemia. However, it remains unknown whether OSAHS is an independent influencing factor for the lipid metabolism disorder or what is the relationship between OSAHS and hyperlipidemia. Some researchers have postulated that the OSAHS patients also suffer from the blood glucose metabolism disorder and non-insulin-dependent diabetes mellitus, which might result in hyperglycemia and subsequent hyperlipidemia due to energy conversion [28].

It was found that OSAHS patients also have insulin resistance, which might lead to an increase in CHOL and TG and a decrease in HDL-C. Auto-continuous positive airway pressure treatment is so far the safest and most effective therapy for moderate and severe OSAHS. Sleep hypoxia may be alleviated by applying air pressure to eliminate upper airway collapse, adjust sleep habits, and result in improvement of OSAHS symptoms. Our findings also indicate that auto-continuous positive airway pressure could effectively decrease hyperlipidemia in obese OSAHS patients. The symptoms of daytime sleepiness, fatigue, and night awakenings due to shortness of breath, dizziness, and dry mouth upon awakening in the morning were all significantly alleviated with treatment; the AHI, mean nocturnal SaO_2_, and minimum SaO_2_ all significantly improved. The serum level of TG and HDL-C was significantly reduced after auto-continuous positive airway pressure treatment, while there was no significant change in the BMI, neck circumference, and waist hip ratio. These findings further indicate that there may be a relationship between the blood lipid metabolism disorder and OSAHS, which was independent from obesity. The serum gamma-glutamyl transpeptidase (GGT) improved after auto-continuous positive airway pressure treatment but not significantly, which might be due to the short treatment period in the present study. Although it is already known that OSAHS is correlated with hyperlipidemia and insulin resistance, there was no significant difference between the two groups in terms of CHOL and LDL-C. However, significant differences were found with respect to TG and HDL-C.

### The effect of OSAHS on serum ApoM and ApoA5

In the current study, a positive correlation between serum ApoM and ApoA5 and HDL was observed, indicating that OSAHS might be involved in lipid metabolism disorder by regulating ApoM and ApoA5 on HDL. However, this speculation is in need of further confirmation.

Meanwhile, the positive correlation between serum ApoM and ApoA5 and HDL indicates that ApoM and ApoA5 might be the apolipoproteins contributing to HDL abnormality in OSAHS, which is in accordance with previously published data [29].

The serum levels ApoM and ApoA5 in the OSAHS group were significantly lower in comparison to the control group after age, gender, and BMI parameters were matched. This finding indicates that the more severe the OSAHS, the lower the serum levels of ApoM and ApoA5. In the present study, auto-continuous positive airway pressure treatment lowered serum ApoM, ApoA5, and HDL levels significantly, further confirming the correlation between OSAHS severity and the serum levels of ApoM and ApoA5.

Multivariate regression analysis indicated that the serum ApoM and ApoA5 levels are correlated with mean SaO_2_, which reflects the severity of OSAHS. Serum ApoM, ApoA5, and HDL levels in OSAHS patients after treatment strongly indicated that ApoM and ApoA5 are the important bridge between OSAHS and HDL. Given the high mortality rate and severe adverse effects on organs, OSAHS has gradually gained importance in the health care field. There are many common features between OSAHS and hyperlipidemia—both are common diseases strongly associated with obesity, aging, glucose metabolism disorder, and hypertension. OSAHS is usually concurrent with hyperlipidemia, although it has been found that OSAHS and its related hypoxia could cause the lipid metabolism disorder independently from obesity [30].

This study has some limitations. As a retrospective, cross-sectional study, there may be some bias in the results. Therefore, further study is needed to fully investigate the cause and effect of cardiovascular problems in OSAHS and whether these are directly related to lipid metabolism disorder. The number of included subjects was low and included many more males than females; this gender difference in the population is likely to be influenced by males having an increased likelihood of having OSAHS [23, 24].

In the present study, the serum HDL levels decreased in OSAHS patients along with serum ApoM and ApoA5 levels, indicating that ApoM and ApoA5, which are involved in the HDL disorder, may also be regulated by intermittent hypoxia. This new finding may help facilitate an in-depth study on OSAHS and hyperlipidemia and open up a new therapeutic option.

## Abbreviations

AHI: Apnea Hypopnea Index
ApoA5: Apolipoprotein A5
ApoB: Apolipoprotein B
ApoM: Apolipoprotein M
BMI: Body mass index
CHOL: Total cholesterol
DBP: Diastolic blood pressure
ECG: Electrocardiogram
EEG: Electroencephalogram
EMG: Electromyography
EOG: Electromyogram
HC: Hip circumference
HDL: High-density lipoprotein
LCAT: Lecithin cholesterol acyltransferase
LDL: Low-density lipoprotein
Lpa: Lipoprotein a
LSaO2: Lowest oxygen saturation
MSaO2: Mean oxygen saturation
NC: Neck circumference
OSAHS: Obstructive sleep apnea hypopnea syndrome
PSG: Polysomnography
SBP: Systolic blood pressure
TG: Triglyceride
T-SaO_2_ < 90%: Percentage of blood oxygen saturation below 90 % during sleep
VLDL: Very low-density lipoprotein
WC: Waist circumference
WHR: Waist hip ratio
